# An Incremental Mori-Tanaka Homogenization Scheme for Finite Strain Thermoelastoplasticity of MMCs

**DOI:** 10.3390/ma3010434

**Published:** 2010-01-13

**Authors:** Heinz E. Pettermann, Christopher O. Huber, Mathias H. Luxner, Sergio Nogales, Helmut J. Böhm

**Affiliations:** 1 Institute of Lightweight Design and Structural Biomechanics, Vienna University of Technology, Gusshausstr. 27-29, A-1040 Vienna, Austria; E-Mail: hjb@ilsb.tuwien.ac.at; 2 Austrian Aeronautics Research (AAR), Network for Materials and Engineering, Austria; 3 Luxner Engineering ZT, Christian Plattner-Str. 4, A-6460 Imst, Austria

**Keywords:** constitutive material law, thermoelastoplasticity, finite strain, incremental mean field scheme, multiscale analysis, matrix/inclusion composites, structural analysis

## Abstract

The present paper aims at computational simulations of particle reinforced Metal Matrix Composites as well as parts and specimens made thereof. An incremental Mori-Tanaka approach with isotropization of the matrix tangent operator is adopted. It is extended to account for large strains by means of co-rotational Cauchy stresses and logarithmic strains and is implemented into Finite Element Method software as constitutive material law. Periodic unit cell predictions in the finite strain regime are used to verify the analytical approach with respect to non-proportional loading scenarios and assumptions concerning finite strain localization. The response of parts made of Metal Matrix Composites is predicted by a multiscale approach based on these two micromechanical methods. Results for the mesoscopic stress and strain fields as well as the microfields are presented to demonstrate to capabilities of the developed methods.

## 1. Introduction

The use of composite materials in components and parts often requires structural analyses of these parts during the design process. The desired simulation tools should reliably predict the behavior of the structure as well as of the material. These needs go far beyond materials characterization, *i.e.*, giving the response of specimens to selected load cases. To perform structural analyses of components constitutive material laws are essential. They must be capable of handling any loading scenario which may occur in a part under service conditions at each local material point.

A very universal tool for structural analysis is the Finite Element Method (FEM) by which the response on the macroscopic length scale can be treated. Employing constitutive material descriptions which are based on micromechanics gives access to the smaller length scales. Since Metal Matrix Composites (MMC) are to be dealt with, thermoelastoplastic material response is considered in the finite strain regime.

Computational tools employed in such micromechanical predictions are challenged by numerous variables associated with the composition of the material, e.g., type, volume fraction, sizes, shapes, or distributions of reinforcing particles. These affect the thermomechanical and physical properties of MMCs, such as thermoelastic, elastoplastic, or conductive properties. On the one hand, modeling tools have to be capable of reliably predicting the overall response of the composite based on the input of the individual properties of the phases. On the other hand, they have to correctly translate prescribed macroscopic loads into local responses. The above procedures are referred to as homogenization and localization, respectively.

There are two widely used approaches in micromechanics of materials. Mean field methods aim to obtain the overall properties of inhomogeneous materials in terms of phase averages of the stress and strain fields. They usually give rise to analytical (or semi-analytical) procedures, and, as a consequence, pose low computational requirements. They are typically based on Eshelby’s solution [1] for a single ellipsoidal inclusions in an infinite matrix. For non-dilute inclusions, Mori-Tanaka type methods [2,3,4] allow an efficient evaluation of the thermoelastic properties of MMCs covering a wide variety of inclusion shapes and distributions. Higher order methods have been developed on the basis of statistical considerations [5] which give better predictions for the stress fields and the effective properties.

When investigating inhomogeneous materials containing thermoelastoplastic constituents, there have been two main lines of development. One the one hand, the secant plasticity concept has been applied [6,7] which is limited to radial loading paths in stress space. On the other hand, incremental plasticity models, such as the incremental Mori-Tanaka (IMT) method, have been developed [8] for small strain applications. An essential feature of the IMT is the capability of accounting for non-radial load paths, an important prerequisite for its use as a constitutive law. In the elastoplastic regime, the IMT typically overestimates overall strain hardening of particulate MMCs, but recently improvements have been achieved by modifications to the algorithms [9,10].

The second micromechanical approach involves the evaluation of highly resolved local fields of a specific microstructure by employing discrete models of its geometry. Periodic microfield approaches (PMAs) typically employ unit cells to study periodic phase arrangements in order to limit the computational effort [11,12]. However, high resolution discretization of unit cells containing a number of randomly positioned particles in a matrix results in large models as well as considerable computation times. Consequently, a procedure based on the PMA using unit cells is not suited to predicting the response of an entire component, but is typically employed for computational material characterization. Second order PMA-type approaches have been developed [13] which take into account moments and rotations of the unit cells. They also have been introduced at each Gauss point in FEM simulations to give the constitutive response. However, three-dimensional structural analyses based on three-dimensional unit cells seem to be far out of reach with today’s simulation hardware.

In this work, both micromechanical approaches for performing homogenization and localization are combined and employed within the framework of FEM. The IMT method is extended to account for finite strains [14,15], and is used to predict the effective MMC response. The PMA, operating at finite strains, too, is employed to investigate the local fields in the matrix and reinforcements, and, by homogenization, the effective response on the mesoscale. These methods are employed at appropriate lengths scales to enable structural analysis of MMC components, taking into account the matrix/inclusion interaction explicitly. For the MMC full three-dimensional analyses are carried out always, which is particularly necessary for adequately capturing the plastic flow of the matrix material [16].

The quality of the results of the analytical IMT approach as well as its limit of applicability are evaluated by comparing results with predictions from periodic unit cell models.

## 2. Modeling Approach

A hierarchical concept is used, by which the material as well as the component responses are investigated on different length scales. These are defined as the *macro*scale of the MMC component, the *meso*scale at which the MMC is regarded as homogenized material, and the *micro*scale which is set by the inclusions’ size. On all scales, constitutive formulations are utilized which involve co-rotational Cauchy stresses. Logarithmic strains with additive decomposition of elastic and plastic contribution are used, since metal plasticity is to be investigated.

### 2.1. Thermo-elastic-plastic mean field approach

In the following sections derivations are given for the case of linear thermo–elastic inclusions embedded in a thermo–elasto–plastic matrix material. It should be noted at this point that equivalent formulae can also be obtained for thermo–elasto–plastic inclusions in an thermo–elastic matrix and an equivalent implementation may be used (which then allows the application to approximate analysis of functionally graded materials (FGM) [16,17,18]).

The derivation of the IMT for small strains follows [8] and is given here in terms of strains. Note that equivalent equations hold true for stress as well. The micro scale averaged strain rate tensor, $d\varepsilon^{\left(p\right)}$, of each phase (p) is related to the meso scale far field strain rate tensor, $d\varepsilon_{\mathrm{mech}}$, by the instantaneous strain concentration tensor, ${{\bar{A}}_{t}}^{\left(p\right)}$. Thermo-mechanical effects, which are due to the thermal expansion mismatch of the constituents, can be accounted for by introducing the instantaneous thermal strain concentration tensor, ${\bar{a}}_{t}^{\left(p\right)}$. For a given homogeneous temperature rate, $d\bar{\vartheta }$, this thermal concentration tensor allows for the computation of the phase thermal strain rates. The total strain rates in each phase (p) can then be expressed as

(1) $$d\varepsilon_{tot}^{\left(p\right)}={\bar{A}}_{t}^{\left(p\right)}\;d\varepsilon_{\mathrm{mech}}+{\bar{a}}_{t}^{\left(p\right)}\;d\bar{\vartheta }\;$$

At this point the mesoscopic instantaneous mechanical and thermal properties are given in a formulation using the inclusion volume fraction, *ζ*, and the matrix strain concentration tensor, ${\bar{A}}_{t}^{\left(m\right)}$, as

(2) $$E_{t}=E^{\left(i\right)}+\left(1-\zeta \right)\left({E_{t}}^{\left(m\right)}-E^{\left(i\right)}\right){\bar{A}}_{t}^{\left(m\right)}\;$$

(3) $$\alpha_{t}={\left(E_{t}\right)}^{-1}\left[e^{\left(i\right)}+\left(1-\zeta \right){\left({\bar{A}}_{t}^{\left(m\right)}\right)}^{T}\left({e_{t}}^{\left(m\right)}-e^{\left(i\right)}\right)\right]\;$$

where $E^{\left(i\right)}$ is the elasticity tensor of the inclusion material, ${E_{t}}^{\left(m\right)}\left(=d\sigma_{tot}^{\left(m\right)}/d\varepsilon_{mech}^{\left(m\right)}\right)$ is the instantaneous elasto–plastic tangent tensor of the matrix material which has to be updated to actual values in the course of evaluating the matrix behavior by an incremental plasticity law. Note that the relations ${e_{t}}^{\left(m\right)}=-{E_{t}}^{\left(m\right)}\alpha^{\left(m\right)}$ and $e^{\left(i\right)}=-E^{\left(i\right)}\alpha^{\left(i\right)}$ are used in the present context and the phases are represented by the superscripts (m) in case of the matrix and (i) in case of the inclusions, respectively. The transpose is denoted by ${}^{T}$.

In the present work the Mori–Tanaka method is employed which accounts for inclusion interaction in an averaged sense but not in the sense of pairwise interactions. The instantaneous matrix concentration tensors are evaluated by the formulation of Benveniste [3] as,

(4) $${\bar{A}}_{t}^{\left(m\right)}={\left[\left(1-\zeta \right)I+\zeta {\left[I+S_{t}{C_{t}}^{\left(m\right)}\left(E^{\left(i\right)}-{E_{t}}^{\left(m\right)}\right)\right]}^{-1}\right]}^{-1}\;$$

(5) $${\bar{a}}_{t}^{\left(m\right)}=\left(I-{\bar{A}}_{t}^{\left(m\right)}\right){\left(E^{\left(i\right)}-{E_{t}}^{\left(m\right)}\right)}^{-1}\left({e_{t}}^{\left(m\right)}-e^{\left(i\right)}\right)\;$$

Accordingly, they are functions of the inclusion volume fraction, the elastic and instantaneous elasto–plastic material tensors of the phases, and the instantaneous Eshelby tensor, $S_{t}$. The latter is a function of the inclusion aspect ratio and the instantaneous material properties of the matrix phase. In the elasto–plastic regime the instantaneous tensor which describes the matrix material has an “anisotropic” structure and, thus, $S_{t}$ has to be calculated numerically, e.g., by numerical quadrature as proposed by Gavazzi and Lagoudas [19].

For aligned fiber reinforcements this approach works quite well. For typical particle reinforced MMCs, however, it severely overestimates the effective strain hardening. Substantial improvements of the predicted hardening can be achieved by isotropization of the instantaneous matrix material tensor [9,10]. Additionally, this modification allows for a straightforward algebraic computation of the Eshelby tensor, see e.g., [20].

#### Finite Strains

The IMT as given in the previous section is based on small strain theory. In the following the considerations are extended to capture finite strains in particulate MMCs. Since metallic materials are involved the treatment is based on co-rotational Cauchy stresses and logarithmic strains with additive decomposition. In general cases of finite elastoplastic deformations for which the directions of the principal stretches do not rotate simultaneously with the material basis, the rate of logarithmic strain cannot be integrated to form a proper strain measure, however, an incremental approach can be given which is appropriate for implementation into FEM [21]. The increment of the deformation gradient reads,

(6) ΔF=ΔVΔR

where ***V*** is the stretch in the current configuration and ***R*** is the rotation of the material basis. For metals is it common to use logarithmic strains the increment of which can be derived [21] as,

(7) Δε=lnΔV

The original implementation of the Incremental Mori-Tanaka method [8] is already capable of handling logarithmic strains and Cauchy stresses, but is limited to strain histories for which the material basis does not undergo any rotations. The incorporation of the incremental rotations, ΔR, requires two steps. First, in terms of the implementation, all tensors describing states have to be updated according to the rotation, in particular the strains, stresses, as well as the tangent operator. Second, in terms of the micromechanical derivation of the IMT method, the localization relation has to be extended. In the original version the implementation is based on Equation (1). For incorporation of the rotations of the material basis, *i.e.*, to obtain a complete set of localization equations, relations between the mesoscopic rotation and the mean field rotations of the individual phases are required. Since particle reinforced composites are under consideration it is assumed that,

(8) $$\Delta R={\Delta R}^{\left(m\right)}={\Delta R}^{\left(i\right)}\;$$

*i.e.*, the “rotation concentration tensors” are approximated by the identity tensor. In other words, the phase rotations are assumed to be equal (in the mean field sense). This implies that the rotation of the matrix phase is fully conveyed to the embedded linear elastic particles.

Beyond the assumption on the rotations (which will be justified later), the general limitations of first order mean field models apply. Matrix plasticity is relatively “well behaved” and does not kick in self-accelerating mechanisms but evens out peak values in the microstressfields. In contrast, particle or interface fracture would be much more difficult to treat properly by mean field models.

Some shortcomings may be accepted in view of the tremendous advantage of having a constitutive model at hand, which is fully employable in the framework of structural analyses of matrix/inclusion type composite components.

#### FEM implementation

The approach is implemented into the Finite Element Method software *ABAQUS/Standard (Dassault Systémes Simulia Corp., Providence, R.I., USA)* as constitutive material law accounting for temperature dependent material parameters. For the matrix material J2 plasticity with isotropic hardening is used. Equilibrium for the material response is solved for by an implicit Euler backward scheme also taking care of an appropriate formulation of the tangent operator. Special considerations have to be used for the computation of the coefficients of thermal expansion of the MMC which are functions of the instantaneous (elastoplastic) response of the matrix material [8]. Thus, they cannot be given beforehand but have to be evaluated and updated permanently in the course of the nonlinear analysis for each Gauss point.

The extension from the small strain formulation to finite strains entails the extension of the matrix constitutive behavior to finite strain J2 metal plasticity and the handling of the rotations of the material bases. Within the chosen framework, finite plastic deformations are associated with rotations of the material bases at the micro- as well as the mesoscales.

### 2.2. Numerical unit cell approach

For the PMA, a cuboidal multi-particle unit cell is taken from [22]. Some twenty, non-percolating spherical particles of equal size are distributed randomly. The master node concept is utilized to apply mesoscopic deformations in terms of the deformation gradient. As mesoscopic responses, the master nodes’ reactions are evaluated in terms of co-rotational Cauchy stresses. Evaluation of the highly resolved microfields is performed statistically and described in terms of histograms.

In finite strain homogenization of PMA care has to be taken by applying the “push forward” and “pull back” relations. In general, these do not hold true for mesoscopic fields from volume averaging since the co-rotational quantities in the unit cell are not (necessarily) related to a unique coordinate system.

### 2.3. Hierarchical multi scale approach

Both approaches are combined to make use of their individual capabilities and advantages. The first step in applying the hierarchical concept is the prediction of the response to overall loading by the FEM, employing the IMT as mesoscopic constitutive material law. Due to its low computational requirements, the IMT is particularly suited to this task. Furthermore, localization from the meso- to the microscale is performed by this method in terms of phase averages on the microscale. The second step is accomplished by selection of appropriate locations in the macroscopic model, extraction of the evolution of the mesoscopic deformation gradients with overall loading, and application of these deformation histories as mesoscopic loads to the computationally expensive PMA. On the one hand, this procedure gives access to the mesoscopic MMC responses, which are used to verify the IMT results attained in the previous step. On the other hand, the microfields of the PMA are predicted for detailed insight into the matrix and particle scale. Additionally, they are compared to the IMT’s phase averaged results to assess the latter’s limit of applicability.

## 3. Example

**Figure 1:**
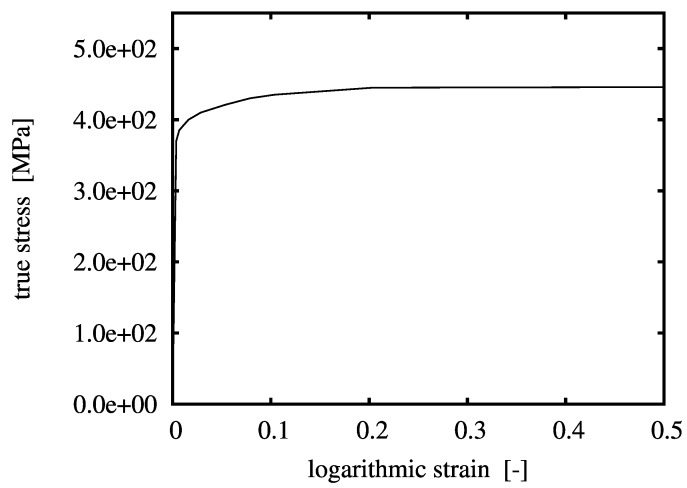
Uniaxial stress-strain curve of the matrix material.

Simulations are performed on a Gleeble-type experiment, which is a compression test of a cylindrical specimen between two anvils. The length and diameter of the specimen are 15 mm and 10 mm, respectively. The model material investigated in this work is an MMC with 20 vol % of ceramic particulate inclusions. The particles are assumed to be of spherical shape and statistically homogeneously distributed. The matrix is taken to behave elastoplastically and the inclusions are linear elastic; both phases are isotropic. The Young’s modulus and the Poisson ratio of matrix and particulate inclusions are $E^{\left(m\right)}=100$GPa, $\nu^{\left(m\right)}=0.3$, $E^{\left(i\right)}=400$GPa, $\nu^{\left(i\right)}=0.19$, respectively. The flow curve of the matrix material is shown in Figure 1. Associated J2-plasticity and isotropic hardening are adopted. A perfect interface bonds the individuals phases and no damage is assumed to occur throughout the loading history.

**Figure 2:**
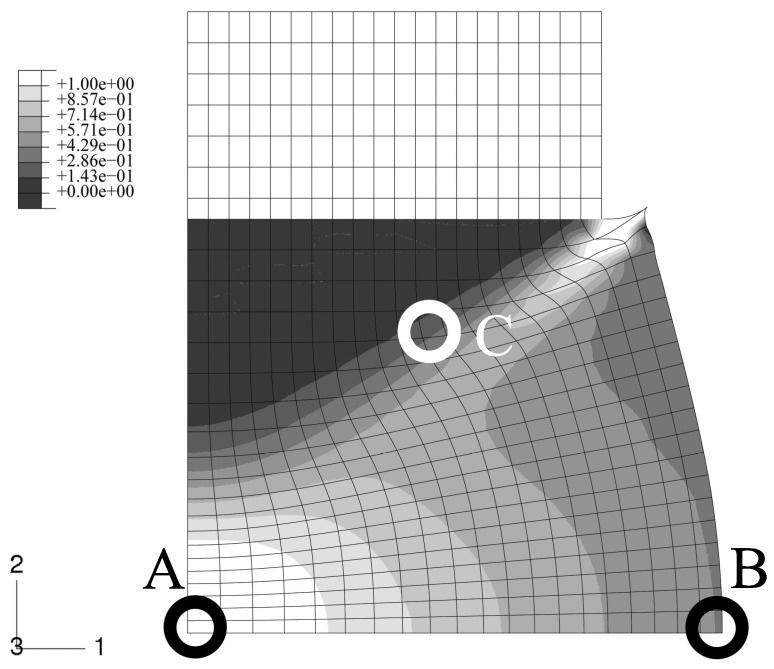
FEM simulation of the macroscopic uniaxial compression of a cylindrical MMC component; undeformed regular axisymmetric mesh and true scale deformation; contours show the accumulated equivalent plastic in the matrix phase; selected locations A,B,C for subsequent detailed analyses.

An axisymmetric FEM model for the upper half of the specimen is employed using continuum elements with biquadratic displacement interpolation and reduced integration. The initial aspect ratio of the elements is set to 2/3 (radial/axial dimension). In Figure 2, the undeformed, homogeneous mesh is visible at the top. The global deformation applied resembles a reduction in length to 2/3, and is prescribed in the axial direction at the top edge. To model stiction between specimen and anvil, radial displacements at this edge are locked.

The FEM simulation of the above sample is carried out with the constitutive behavior of the MMC described by the IMT. Three locations in the macroscopic model are chosen such that a broad spectrum of mesoscopic stress and strain histories is provided for detailed analysis by the PMA (Figure 2). Location A is the Gauss point closest to the center of the specimen. Location B is close to the outer surface, and location C represents a region where shear deformation is marked.

### 3.1. Results

#### Macro response

The final state of deformation is described by the macroscopic deformation gradient in axial direction, $F_{22}=2/3$. The predicted global deformation of the specimen in this state is shown in Figure 2; no deformation scaling is used. The stiction condition between the anvils and the specimen causes barreling. Note that elements at the top right corner are severely distorted because of the chosen load introduction. However, this region is not evaluated, and minor influence on the response of the other regions is assumed. No FEM convergence problems were caused, which shows the robustness of the IMT constitutive law and its implementation. The center region of the specimen undergoes the highest deformation. Deformation is constrained at the top, where a cone-shaped region of moderately deformed material is driven through the sample.

#### Mesoscopic response

**Figure 3:**
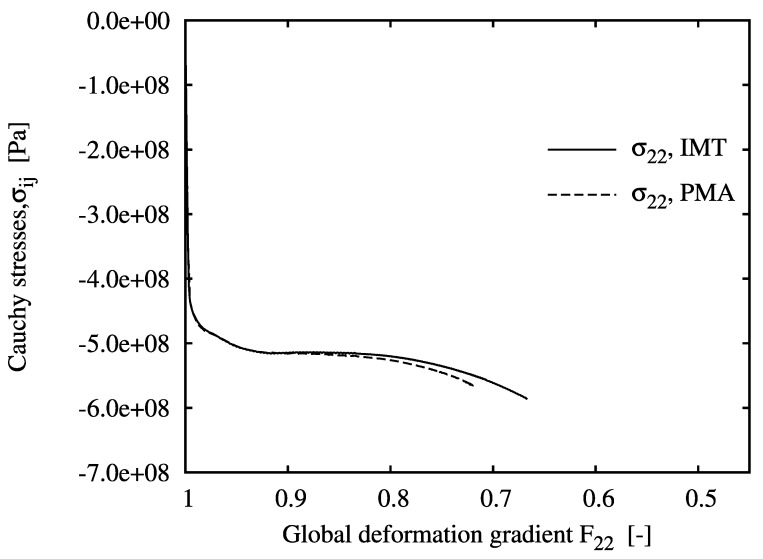
Evolution of the mesoscopic Cauchy stress tensor component $\sigma_{22}$ over the global deformation gradient component $F_{22}$ for location A, predicted by the IMT and the PMA.

The evolution of the mesoscopic Cauchy stresses at the selected locations versus the global measure of deformation, $F_{22}$, is shown in Figure 3 to Figure 5. The figures include the IMT predictions as well as the unit cell results, which agree excellently for all cases. For location A, the mesoscopic stress histories resemble an almost purely uniaxial compressive stress state, since this point lies almost at the axis of rotational symmetry. Some `hardening’ is shown despite the nearly ideal plastic flow behavior of the matrix material. Note that for the PMA prediction, no convergence could be achieved beyond a macroscopic deformation gradient of $F_{22}=0.72$.

For location B, a biaxial stress state with compression in the axial and tension in the hoop directions is predicted. Although axial compression is dominant at the macro level, the meso-stress magnitude in this direction reaches a maximum and then decreases in the course of deformation, whereas the tensile hoop stress increases steadily. This markedly non-radial loading history is consistently predicted by the IMT, owing to its incremental formulation. Region C shows a more general stress state including mesoscopic shear. For the present stress-strain formulation, this causes rotation of the material basis. Again, the predictions agree very well, as for the other cases. This demonstrates the excellent predictive quality of the analytical micromechanics based IMT for general loading conditions as compared to the much more costly numerical PMA approach. The final deformation states of the unit cell pertaining to the three locations are displayed in Figure 6. Note that the displacements are shown in real scale.

**Figure 4:**
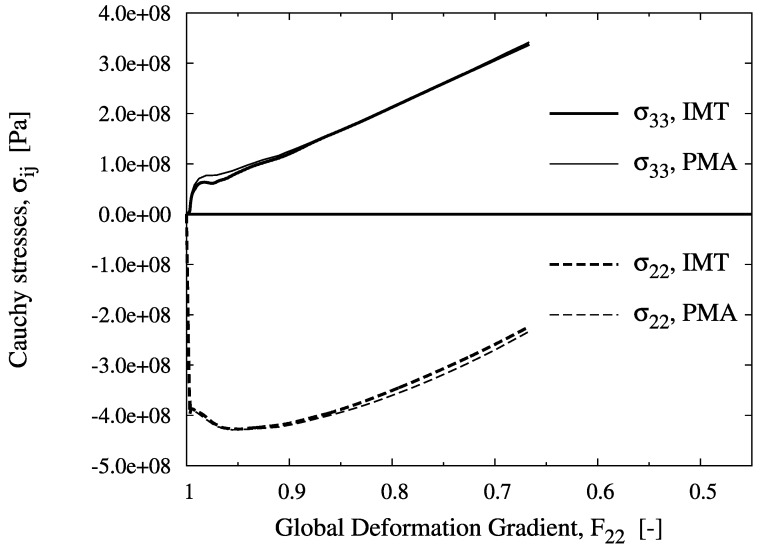
Evolution of the mesoscopic Cauchy stress tensor components over the global deformation gradient component $F_{22}$ for location B, predicted by the IMT and the PMA.

**Figure 5:**
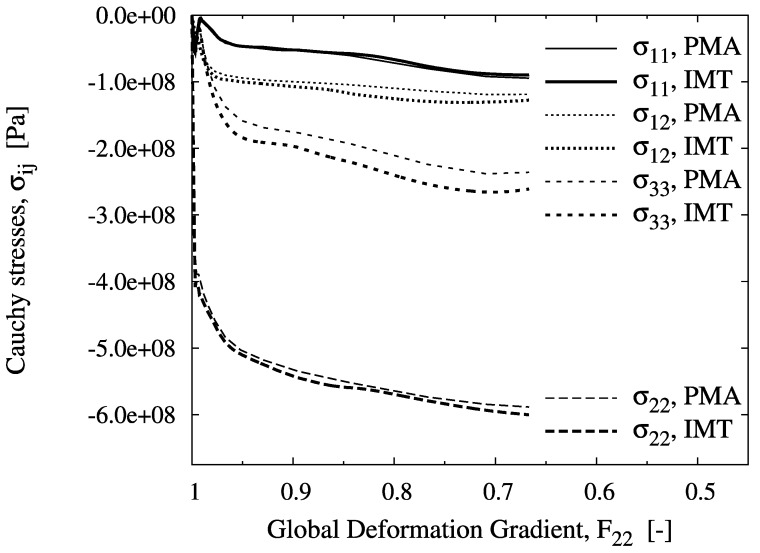
Evolution of the mesoscopic Cauchy stress tensor components over the global deformation gradient component $F_{22}$ for location C, predicted by the IMT and the PMA.

**Figure 6:**
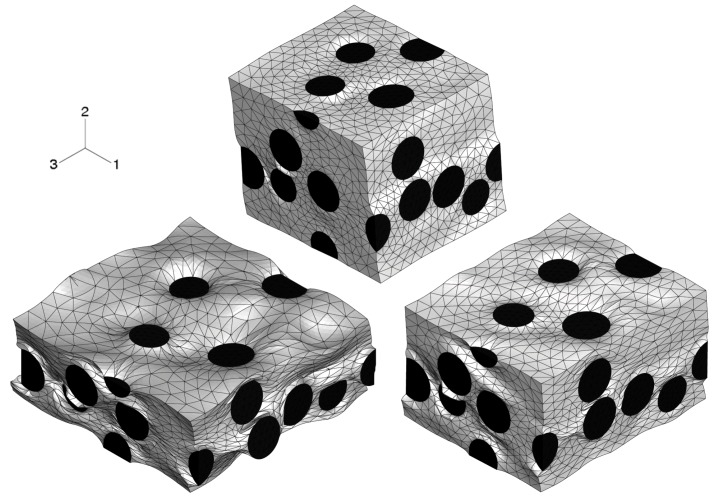
Deformations of the unit cell for the three selected locations A (left), B (right), and C (top), at their final state of deformation, at true scale; undeformed unit cells are cube shaped with random distribution of equi-sized spherical inclusion.

#### Microscopic response—Accumulated equivalent plastic strain in the matrix

**Figure 7:**
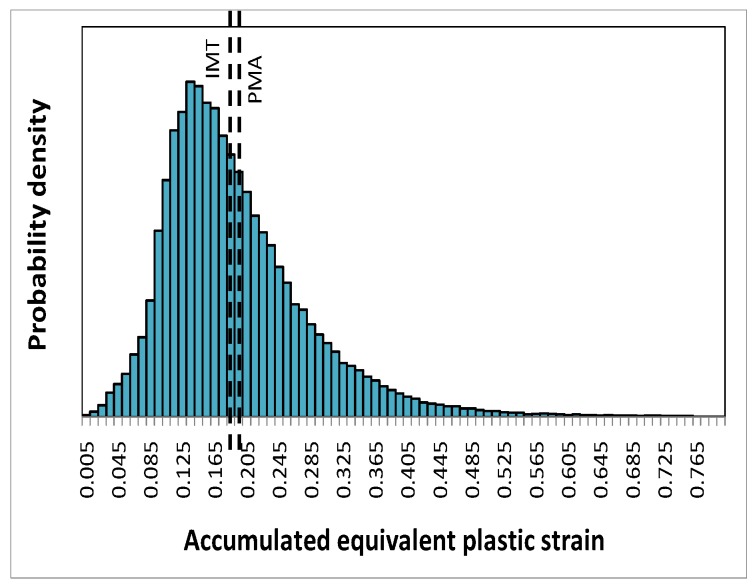
Probability density distribution of the accumulated equivalent plastic strain in the matrix phase as predicted by the unit cell simulations at $F_{22}=0.88$ for location A, the resulting phase average value (PMA), and the IMT prediction.

The probability density distribution of the accumulated equivalent plastic strain in the matrix, as a micro quantity predicted by the unit cell simulations at location A, is shown in Figure 7 for the global deformation gradient $F_{22}=0.88$. Also indicated are its phase averaged value—a meso quantity—evaluated from the unit cell, and the corresponding prediction by the IMT. The latter is very close to the phase average obtained from the unit cell. On the microscale, however, the scatter of the accumulated equivalent plastic strain in the matrix phase is considerable.

#### Microscopic response—Maximum principal stresses in the particles

**Figure 8:**
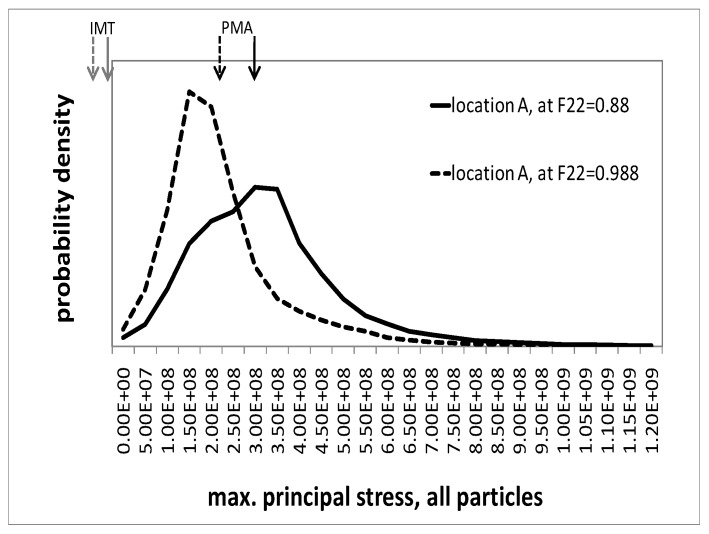
Probability density of the maximum principal stress in all particles as predicted by the unit cell simulations for location A, evaluated at $F_{22}=0.988$ and $F_{22}=0.88$; arrows indicate its mean value (PMA) and the IMT prediction.

**Figure 9:**
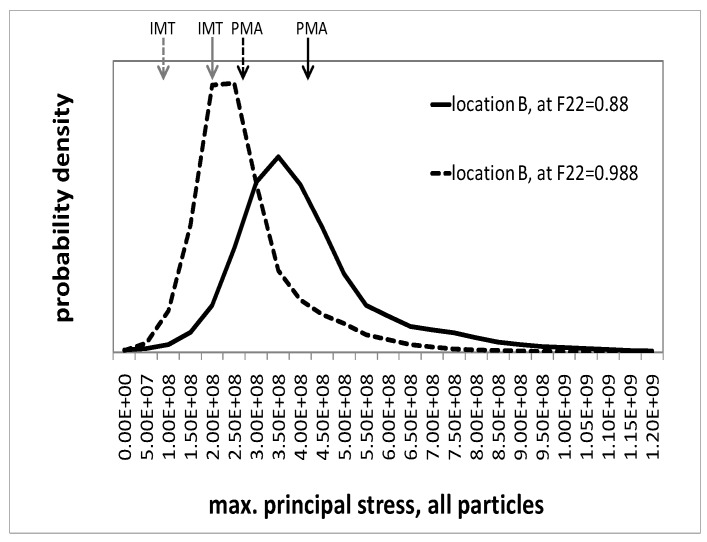
Probability density of the maximum principal stress in all particles as predicted by the unit cell simulations for location B, evaluated at $F_{22}=0.988$ and $F_{22}=0.88$; arrows indicate its mean value (PMA) and the IMT prediction.

The maximum principal stresses can serve as indicators for particle fracture. Figure 8 and Figure 9 show the probability density distribution of the maximum principal stresses in the particle phase, as predicted by the unit cells, for locations A and B, respectively. Two states in deformation history are illustrated, described by macroscopic deformation gradients $F_{22}=0.988$ and $F_{22}=0.88$, respectively. The figures also show the phase averages from the unit cell and the mesoscopic IMT predictions. It should be noted that the maximum principal stresses at some Gauss points are outside the displayed interval. However, the associated volumes are two orders of magnitudes smaller than that of a particle. Generally, for both selected regions, the distribution of the maximum principal stresses in course of the deformation shifts towards higher values. At the same time, its scatter increases. Although location A is associated with a mesoscopic uniaxial compressive stress state, the unit cell simulation predicts a considerable level of positive maximum principal stresses in the particles, even at moderate deformation, cf. Figure 8. In fact, particle fracture in the center of Gleeble tests has been found experimentally [23]. The IMT severely underestimates the maximum principal stresses; its predictions are compressive with a value of ${\sigma_{I}}^{\left(p\right)}=-20$ MPa at $F_{22}=0.988$ and close to zero at $F_{22}=0.88$, respectively. Note that, strictly speaking, two different quantities are compared to each other. The IMT takes the phase averaged particle stress tensor (a single tensor) from which the principal stresses are evaluated. In contrast, the PMA evaluation relies on a fluctuating tensor field from which in each location the principal stresses are extracted. This way the field fluctuations fully enter the results presented for the PMA in Figure 8.

#### Microscopic response—Rigid body rotation

**Figure 10:**
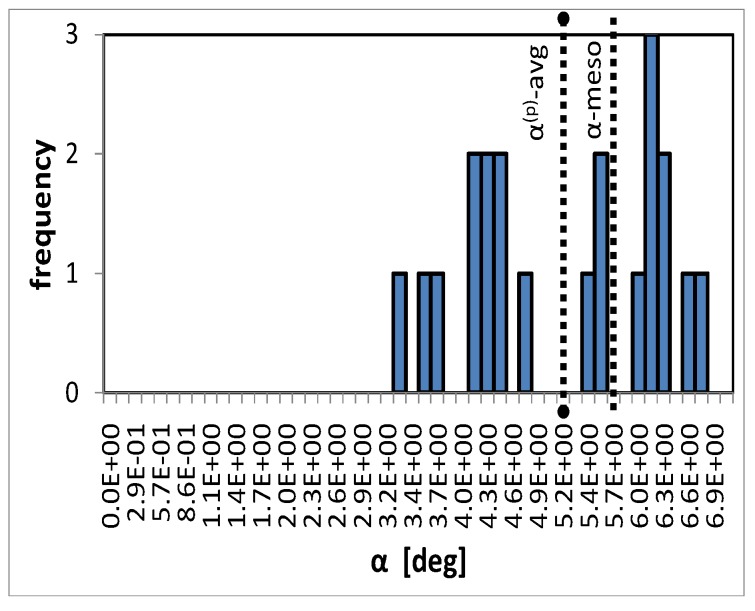
Distribution of average rigid body rotations of individual particles in the 12-plane, the particle phase average $\alpha^{\left(p\right)}$-avg, and the meso scale rigid body rotation due to finite shear deformation, *α*-meso.

Figure 10 shows the distribution of the rigid body rotations of individual particles within the unit cell in the 12-plane for location C. The average rotation of all particles as well as the mesoscopic rotation of the material basis are also indicated in the figure. In this case, the end-state of the simulation is regarded, $F_{22}=2/3$. Rotations of components in other planes than the 12-plane are at least one order of magnitude smaller. Because of the much higher Young’s modulus of the particle phase compared to the stiffness of the matrix, deformations of the particles are small. Therefore, the intra-particle fluctuations within individual particles disappear and the distribution of rotations is represented by the average rotations of the individual particles, cf. 8. The good agreement in the averages as well as the relatively small scatter in rotations of the individual particles justify the assumption made in the finite strain formulation of the IMT.

#### Sample with gradient in composition

**Figure 11:**
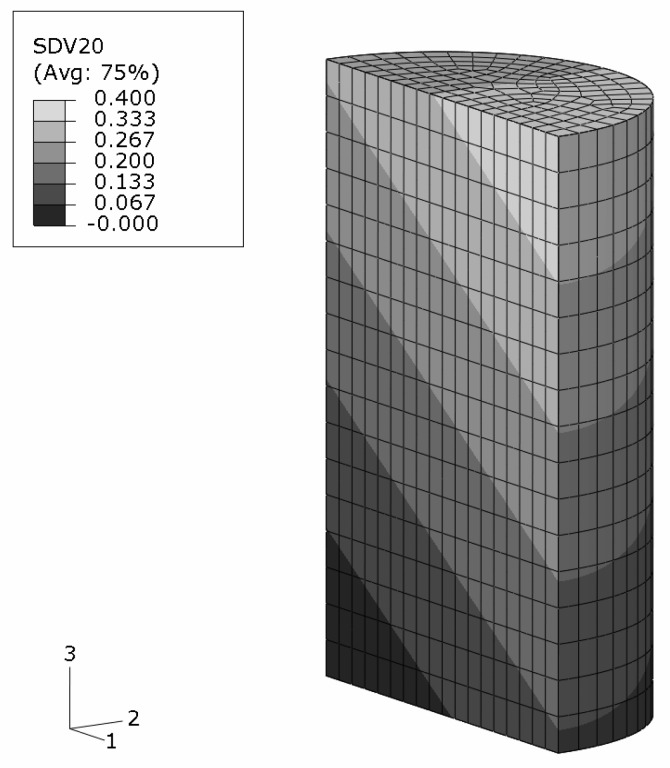
FEM half model of a cylindrical MMC–FGM component with a linear smooth reinforcement volume fraction variation between ζ=0.0 and ζ=0.4.

The above example is modified to account for a non-homogeneous composition. The average reinforcement volume fraction is kept at ζ=0.2 but varies linearly form ζ(x)=0.0 at one bottom edge to ζ(x)=0.4 at the opposite top edge, see Figure 11. The direction of the spatial gradient is inclined at 33.7${}^{\circ }$ with respect to the sample’s cylinder axis so that it runs diagonally through the sample. This requires a three-dimensional FEM analysis where half of the specimen is modeled. Note that the volume fraction is imposed at the Gauss points as a smooth continuous field which does not follow the boundaries of the finite elements. The loading situation and the boundary conditions are kept as in the previous example.

**Figure 12:**
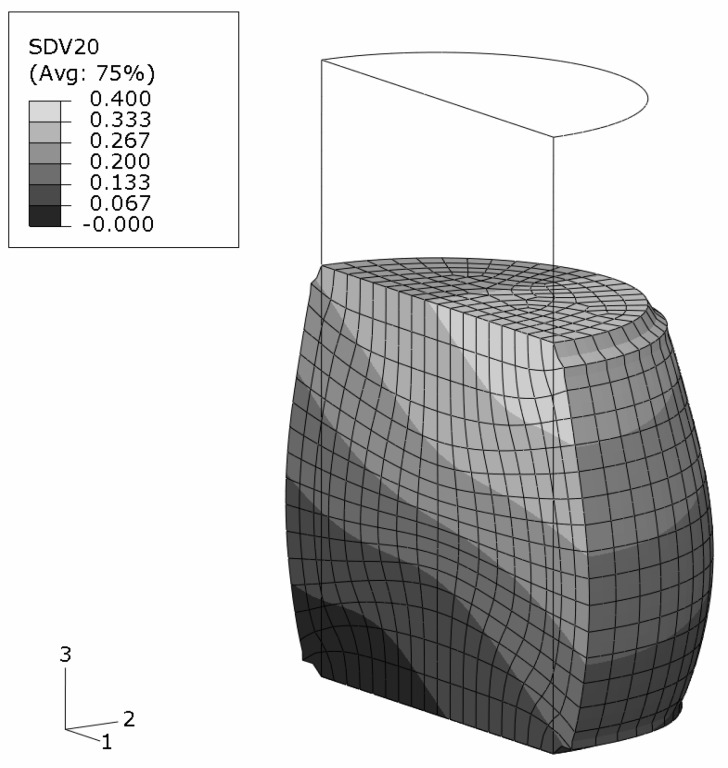
MMC–FGM cylinder under uniaxial compression of $F_{22}=2/3$; reinforcement volume fraction distribution on the predicted deformation in true scale.

The predicted shape of the sample at the global deformation gradient of $F_{22}=2/3$ is shown in Figure 12.

**Figure 13:**
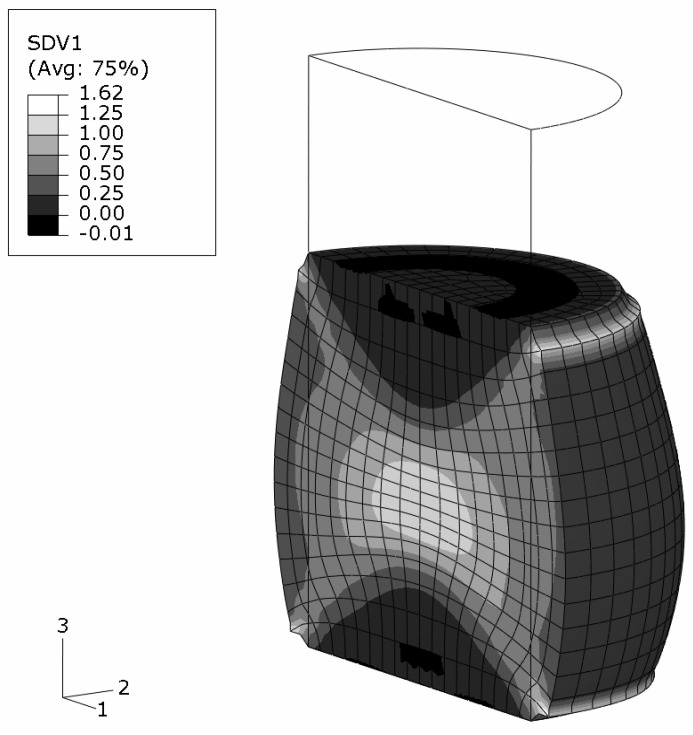
MMC–FGM cylinder under uniaxial compression of $F_{22}=2/3$; predicted accumulated plastic strains in the matrix phase on the true scale deformations (negative strain values arise from extrapolation).

The regions in the vicinity of the top and bottom surfaces are influenced by the applied constraints. The diameter increase is more pronounced for sections with lower reinforcement volume fraction. The skew grading, however, does not show any significant influence on the final shape which is almost axisymmetric. The reason is the distribution of the reinforcement volume fraction in the deformed shape. The originally inclined gradient becomes nearly parallel to the cylinder axis upon deformation, see Figure 12. This also affects the distribution of the accumulated equivalent plastic strain in the matrix phase, Figure 13, for which a minor unsymmetry is predicted. The very limited influence of the MMC’s composition on the elastoplastic behavior of the sample at high macroscopic deformations contrasts the regime of small plastic strains where the symmetry of the equivalent plastic strains is markedly less than in Figure 13.

#### Other load cases (not shown)

Two essential load cases not treated so for are hydrostatic pressure and spatially homogeneous temperature changes. Due to its formulation, the IMT is not capable of predicting plastic yielding in these cases [15]. However, unit cell simulations of these load cases have shown extremely small mesoscopical residual strains, at least at reasonable loads. Consequently, the IMT maintains its good predictive capabilities on the mesoscale.

All numerical tools are fully prepared to include the thermal expansion, temperature history as well as the temperature dependence of the material properties in the simulations.

Note that although the adopted flow curve of the model material almost approaches that of an ideally plastic material, the IMT is capable of utilizing all kinds of hardening curves, including softening (where, however, mesh dependencies occur if straightforward FEM techniques are applied). Several tests were performed successfully using strain hardening as well as strain softening [15].

## 4. Conclusion

Micromechanics based approaches are presented which allow for structural analyses in the finite strain regime of parts and components made of matrix/inclusion type composites. An analytical incremental Mori-Tanaka (IMT) scheme for the prediction of the thermoelastoplastic behavior is extended to account for finite strains. It is implemented as a constitutive material law into Finite Element software in an Euler backward manner and accounts for arbitrary loading scenarios as well as temperature dependent material data. It is employed to predict mesoscale responses and approximations of microscale fields. A periodic microfield approach (PMA) using unit cells is utilized to predict the meso responses as well as the fluctuating microfields. It also serves as a reference to assess the quality of the results of the analytical approach.

As an example the computational simulation of a forming process of a particulate metal matrix composite component is performed. A hierarchical concept is utilized within the framework of the Finite Element Method to model a Gleeble-type experiment employing two micromechanics of materials approaches. When comparing the IMT results to those of the PMA on the mesoscale, the formers’ predictions are found to be excellent and the assumptions made in the IMT’s formulations are shown to hold.

The following procedure is shown to be successful. In predicting the response of a macroscopic composite component the IMT is employed to provide quick and reliable access to the material response at the mesoscale, where its predictions render excellent results. As the periodic microfield approach is computationally very expensive, it is used for selected regions only if more detail at the fluctuating fields in the phases is necessary, e.g., to provide information about the onset of damage.

The general formulation of the IMT allows the investigation of a variety of problems, including functionally graded materials and structures with spatially changing composition (e.g., reinforcement volume fraction). General thermomechanical loading situations can be simulated as occurring in the production and in the service life of components and parts.
